# Sketching the Power of Machine Learning to Decrypt a Neural Systems Model of Behavior

**DOI:** 10.3390/brainsci9030067

**Published:** 2019-03-20

**Authors:** Monique Ernst, Joshua L. Gowin, Claudie Gaillard, Ryan T. Philips, Christian Grillon

**Affiliations:** 1 Section on Neurobiology of Fear and Anxiety (NFA), National Institute of Mental Health/NIMH, 15K North Drive, Bethesda, MD 20892, USA; ryan.philips@nih.gov (R.T.P.); grillonc@mail.nih.gov (C.G.); 2 Departments of Radiology and Psychiatry, University of Colorado School of Medicine, Aurora, CO 80045, USA; joshua.gowin@ucdenver.edu; 3 IReach Lab, Unit of Clinical & Health Psychology, Department of Psychology, University of Fribourg, 1700 Fribourg, Switzerland; claudie.gaillard@unifr.ch

**Keywords:** triadic neural systems model, development, adolescence, machine learning, networks

## Abstract

Uncovering brain-behavior mechanisms is the ultimate goal of neuroscience. A formidable amount of discoveries has been made in the past 50 years, but the very essence of brain-behavior mechanisms still escapes us. The recent exploitation of machine learning (ML) tools in neuroscience opens new avenues for illuminating these mechanisms. A key advantage of ML is to enable the treatment of large data, combing highly complex processes. This essay provides a glimpse of how ML tools could test a heuristic neural systems model of motivated behavior, the triadic neural systems model, which was designed to understand behavioral transitions in adolescence. This essay previews analytic strategies, using fictitious examples, to demonstrate the potential power of ML to decrypt the neural networks of motivated behavior, generically and across development. Of note, our intent is not to provide a tutorial for these analyses nor a pipeline. The ultimate objective is to relate, as simply as possible, how complex neuroscience constructs can benefit from ML methods for validation and further discovery. By extension, the present work provides a guide that can serve to query the mechanisms underlying the contributions of prefrontal circuits to emotion regulation. The target audience concerns mainly clinical neuroscientists. As a caveat, this broad approach leaves gaps, for which references to comprehensive publications are provided.

## 1. Introduction

Adolescence is a period during which individuals undergo irreversible transformations in multiple physical, biological, cognitive, emotional, social, and behavioral domains. This implies immense complexity in trying to capture the overall landscape of these mutations. This review presents how the use of machine learning (ML) tools could test neural systems theories of motivated behaviors, particularly across the developmental period of adolescence. This is not a tutorial nor a pipeline, but rather an introduction to the growing possibilities that the combination of powerful ML tools with large datasets opens up to probe brain-behavior questions. An effort was made to keep this complex topic as straightforward as possible, at the expense of discussions of limitations and constraints. However, readers are referred to publications that begin to address these gaps.

**Figure 1:**
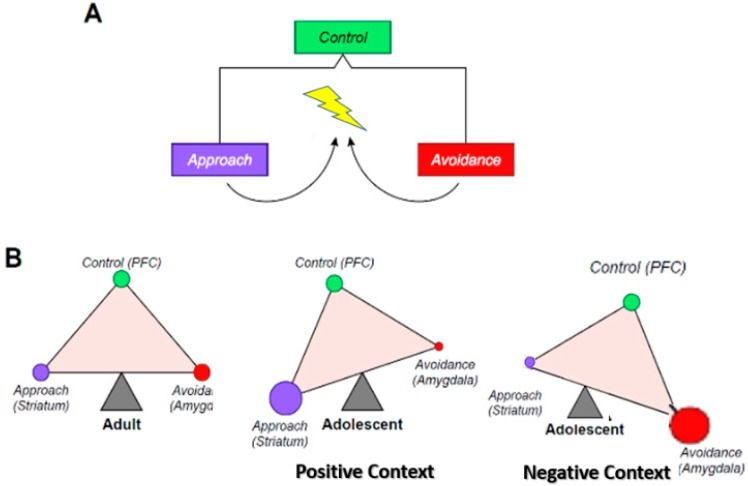
The triadic model. (**A**) This figure shows that at the most elementary level, behavioral responses to a stimulus can take only two forms: approach or avoidance. The selection of the behavioral response is monitored or adjudicated by a supervisory controller (control system). In yellow is the stimulus (i.e., object, situation), purple is the approach response, red is the other possible avoidance response, and green is the controller. (**B**) This represents the neural translation (nodes of the triadic model) of the three behavioral entities described in (**A**). The term node is used here to refer to a neural system whose core structure is unique. The ventral striatum is specific to the approach system, the amygdala to the avoidance system, and the prefrontal cortex (PFC) to the control system. In addition, these three systems establish a balance that is represented as a triangle in equilibrium. The adult balance is used as the yardstick, to which the adolescent is being compared. In an approach context, the adolescent balance is tilted towards the approach system, and away from the avoidance system, in a way that translates the proclivity of adolescent behavior towards approach, including risk-taking. In an avoidance context, the adolescent balance is tilted towards the avoidance system, and away from the approach system, in a way that translates the proclivity of adolescent behavior towards emotional intensity and lability, perhaps reflecting the peak onset of internalizing disorders in adolescence.

At present, only simple heuristic models of the ontogeny of neural systems have been proposed to account for the behavioral changes occurring in adolescence (e.g., [1,2,3]). Among these models, the triadic model [2] figures as the broadest theory that provides functional mechanisms underlying motivated behaviors in general, and specifically across development. The triadic model is based on a functional balance across three neural systems dedicated to (1) approach behavior, (2) avoidance behavior, and (3) control processing (Figure 1). The triadic model, as a whole, has not yet been tested, although individual components have been validated, such as the developmental pattern of the approach system across adolescence [4,5,6]. We believe that the introduction of machine learning (ML) tools to brain-behavior analyses and the emerging availability of big developmental data can test the entirety of the model. This review will illustrate how this could be achieved.

This chapter is divided in two main sections. The first section reviews the triadic neural systems model [2,7]. The second section focuses on the ways to test this model, using a hypothetical study of two large community samples, one of adults and the other of children tested repeatedly from childhood into emerging adulthood. Of note, only functional magnetic resonance imaging (fMRI) data were used to identify neural predictors. In addition, we will not provide details on how to acquire, preprocess, and process the fMRI data. All the neural data used for the analyses were scalar variables of intrinsic functional connectivity (iFC) of regions of interest extracted from resting state scans and regional activations in task-related fMRI scans. The iFC values corresponded to Fisher-transformed Pearson correlations.

## 2. The Triadic Neural Systems Model

The three neural systems of the triadic model consist of an approach, avoidance, and control module. At the most elementary level, motivated behaviors can be reduced to the generation of two possible actions, approach or avoidance, and the decision to adopt one or the other action is ultimately regulated by the control system (Figure 1). The triadic model is a generic heuristic explanation of how input information is processed at the brain neural–systems level to generate a motivated action output. 

### 2.1. Conceptual Definition

We first address the approach and avoidance systems and then the control system.

A fundamental assumption of the model is that the approach and avoidance responses are encoded by two separate, although overlapping, neural systems. The “approach system” is classically associated with positive-value encoding, which underlies reward and motivation functions. The “avoidance system” maps to negative-value encoding, which captures punishment and negative emotion processing. These two “approach” and “avoidance” systems are not just the mirror of one another. They have different properties and behave according to distinct rules. For example, punishments weigh more than rewards. Indeed, economic tasks reveal that the subjective value of a $5.00 loss equates to the subjective value of a $10.00 gain [8]. Another example of these loss/gain differences is the impact of uncertainty onto behavior. Uncertainty, which carries a negative cost, increases avoidance but decreases approach. However, this functional separation of the approach and avoidance systems is far from absolute. Overlaps exist at both behavioral and neural levels.

Accordingly, motivation processing is common to both approach and avoidance systems. In other words, motivation fuels approach and avoidance responses. In fact, “motivated avoidance” is another conceptualization of “active avoidance”, a construct currently under investigation in the field of defensive responses (e.g., anxiety, fear) [9,10,11]. Similarly, emotion takes both negative and positive flavors. Taken together, the approach and avoidance systems bleed into one another, which is reflected at the neural level. 

The third component of the triadic model is the control system, which adjudicates courses of action between the approach and avoidance systems. In the context of this chapter, “control” refers to the formation of preference that is transferred to effector sites. Generally, control processes belong to the cognitive domain. Theories on preference formation are still debated, with two main contenders: (1) a feedforward competition of the representation of options and a resolution favoring the strongest signal [12,13,14,15], and (2) an inhibitory feedback of one representation, leaving the other option to prevail [16,17,18,19]. At this point, these potential mechanisms mediating control have not been considered in the triadic model. 

The triadic model is typically represented as a triangle whose angles denote each system, and the sides denote the connections among the systems (Figure 1). Importantly, the triangle can be tilted in various directions, as a function of the weight (i.e., activity) attached to each system. Theoretically, the tilt could be measured by the location of the center of gravity of the triangle. 

### 2.2. Neural Substrates

#### 2.2.1. Approach System

The approach system is centered on the striatum, a relatively large multiplex structure, whose components include the caudate nucleus, nucleus accumbens, putamen, and pallidum. Broadly, the striatum is an essential contributor to the implementation of goals into actions and plays this critical role through the processing of information along parallel striatal–thalamocortical–striatal loops [20]. Although the bulk of research on the striatum has focused on reward function and motivation [21,22,23], much work in the last two decades has been dedicated to the role of striatal networks in aversive processes [24,25,26,27,28]. While both animal and human data clearly indicate involvement of the striatal circuitry in the processing of aversive stimuli, for instance in active avoidance, the exact mechanism is still unclear. However, it is also well-demonstrated that the recruitment of the striatal circuitry predominates and is more consistent in the processing of appetitive than aversive information [29,30,31]. 

#### 2.2.2. Avoidance System

This system is centered on the amygdala. The amygdala is composed of multiple nuclei with distinct functions, which can be grouped into three compartments: (1) an input compartment, carrying multimodal sensory information; (2) a processing compartment, which integrates and broadly interprets the sensory information coming from the cortex and hippocampus; and finally (3) an output compartment, which dispatches the processed signals to effector agents that code physiological and motor responses. The amygdala input receiver is allocated to the lateral nucleus, and the output dispatcher to the extended amygdala (central nucleus of the amygdala and the bed nucleus of the stria terminalis). The information processing takes place in the basal/accessory basal nuclei. This information processing center has not typically been considered an “output region”, but it does have an important one-way projection to the striatum to inform complex motor programs. The amygdala has raised considerable research aimed at understanding its function in the processing of aversive stimuli, including aversive learning [32,33,34]. Similarly, its implication in reward learning has been well established [33,35,36,37,38,39]. However, it is also important to point out that, like the biased responsivity of the striatum towards appetitive stimuli, the amygdala responds prominently and more consistently to aversive than appetitive stimuli [40,41].

#### 2.2.3. Control System

The control system is centered in the prefrontal cortex (PFC). It falls under the umbrella term of cognitive executive function, which is essential to self-directed behavior. Multiple models have been proposed to describe the various components of executive function (e.g., working memory, inhibition, set shifting), and to map these different components onto brain regions (e.g., [42]). The common denominator across these models is the PFC. Regional specialization of the PFC has been parsed out in various ways, depending on theoretical frameworks, such as, to cite just a few, inferior-lateral versus mid-lateral PFC [43], anterior versus posterior PFC [44], or medial versus left lateral versus right lateral PFC [45]. The triadic model does not specify the putative PFC organization to adjudicate on courses of action. However, the framework most fit to accommodate the control of the direction of action (i.e., the decision proper) consists of a combination of regions that integrate the value and salience tagged to the possible options to decide on, and regions that modulate the weight of these value options. The former set of regions receives information (bottom-up process) and the latter apply the information to direct the course of action (top-down). In other words, the first set of regions receives information from the approach (striatum) and avoidance (amygdala) systems, which is sent then to the second set. The second set of regions modulates the activity of the avoidance and/or approach systems, which provides the signal that is dispatched to effectors which implement the action. This framework has been applied to the pattern of the neural mechanisms involved in the expression of defensive responses [46,47], which we generalize to motivated behaviors at large.

### 2.3. Triadic Model in Adolescence

The foundation of the triadic model is to explain how brain maturational changes underlie the prototypical behavioral changes in adolescence. 

At the behavior level, adolescence is characterized by unique patterns in three domains: peak lifetime period of risk-taking, amplified and labile emotions, and highly context-dependent executive control [48,49,50]. In addition, the adolescent world undergoes a dramatic social reorganization. Accordingly, social processes should be considered as a fourth domain, but it is not yet integrated to the triadic model. Finally, adolescence is a time of vulnerability for psychopathology, as evidenced by a peak rise in the incidence of internalizing, addictive, and psychotic disorders. 

The behavioral shifts across adolescence are modeled as a facilitation of approach behaviors, serving an “exploratory purpose.” Indeed, adolescence refers to the transition period from childhood into adulthood, when the individual moves away from the protective family nest and learns to independently navigate the world, which requires risk-taking and exploration. This behavioral pattern can also reflect blindness or increased tolerance for possible failures. 

According to the triadic neural systems model, the proclivity for exploratory behavior would be supported by an increased reactivity of the approach neural system, which would peak in mid-adolescence and taper down into adulthood. An opposite progression is described for the avoidance neural system in the context of responses to reward, i.e., hypo-responsivity of the avoidance system supposedly to protect exploration. Finally, the adjudication by the control neural system between approach and avoidance is becoming progressively more refined and efficient with age. This elementary description of the developmental dynamics of each unit of the triadic system accounts for the most commonly described changes in the adolescent motivated behavior, i.e., risk-taking and improved cognition [7,51].

Although generally less emphasized, the model also supports exacerbated emotions, positive as well as negative. The avoidance neural system, which contributes most significantly to emotional expression, has been shown to be hyper-responsive to aversive stimuli in adolescents compared to adults (e.g., [52,53,54]). This finding suggests that, in a negative context, the adolescent may react more emotionally than the adult. Therefore, the direction of the developmental trajectory depends on the context in which these systems are called into play. 

This cursory description of how the triadic model is instantiated in adolescence, in a way that can explain typical adolescent behaviors, reveals obvious gaps. For example, it is unclear how the approach (appetitive) system is uniquely affected in aversive contexts in adolescence. The neural delineation of the circuits of each system is only partial. The amount and nature of overlaps among the systems is also unclear, and how these overlaps change with age and with context (appetitive versus aversive) has hardly been addressed. The next section explores how these limitations can be leveraged by the combined use of large datasets and machine learning tools.

## 3. Testing the Triadic Neural Systems Model

### 3.1. Introduction to Machine Learning Tools

The potential benefits of machine learning (ML) tools to facilitate discoveries in neuroscience research have generated huge hope and excitement (e.g., [55,56,57,58]). Indeed, these tools have gained enormous popularity among neuroscientists, particularly clinical neuroimagers, at a time when large datasets are becoming publicly available (e.g., [59,60,61,62,63,64]). Historically, ML tools have been developed to pursue artificial intelligence. As a growing field, ML has diversified into branches within statistics, computer science, and mathematics. The interdisciplinary nature of this field presents challenges when applying ML tools to neuroscience questions, especially in the neuroimaging domain, as it requires substantial expertise in a wide range of domains. For example, engineering, neuroscience, advanced physics, and psychology are each deep and well-developed fields. Achieving mastery of each of them is a daunting task. For this reason, collaborative approaches are highly recommended when applying ML tools to neuroscience research, since it behooves a team to have expertise in each of the domains involved in solving a problem. 

Machine learning consists of algorithms that train computers to learn patterns from arrays of variables. Computer science, statistics, and engineering research have been instrumental to the development of these mathematical tools. Typically, nuanced solutions require large pools of data. As the neuroscience concepts addressed by the triadic model are complex, large datasets will be needed to apply ML tools to its testing. Machine learning consists of automated and iterative computations that promote computer learning of patterns (i.e., models). These patterns can serve to classify data and to provide predictive models. For example, ML is a critical tool in artificial intelligence, the science that trains computers to reproduce human behavior. Deep learning is a sub-specialized area of ML that uses multiple layers of learning, with successive layers using the output from previous layers as the input (e.g., [65]). Deep-learning algorithms have reached new limits in accuracy compared to other methods, and they permit to learn more precise rules when other ML approaches reach a plateau [66,67]. Deep learning typically requires substantially more data than simpler machine learning models, and this presents a limitation of its use in neuroscience, since large neuroscience datasets are rare for psychiatric disorders. 

Finally, ML is divided into “supervised” and “unsupervised” algorithms (e.g., [68]). Supervised ML assumes a ground truth (e.g., patient versus healthy groups; faces versus houses) and trains the computer to use data that will best predict the ground truth. The power of these algorithms lies in the fact that the patterns learned are generalizable to testing datasets (not used to train the model). Some examples of supervised algorithms include multilayer perceptrons (MLP), decision trees, and support vector machines (SVMs). In this manuscript we use decision trees. A more detailed description of this algorithm is included later.

Unsupervised ML is not predicated on a ground truth. It provides training to find reliable patterns in data that can inform the constituents of models. In other words, it attempts to group objects (e.g., brain activation maps) according to their intrinsic properties, as opposed to similarity with some ground truth. These algorithms could also be used to identify important dimensions or components of a dataset. Popular unsupervised algorithms including clustering algorithms such as k-means clustering, hierarchical clustering, Gaussian mixture models (GMMs), and dimensionality reduction tools such as principal component analysis (PCA), and independent component analysis (ICA). 

Deep learning is particularly well-suited for these applications. In all cases, ML’s initial solutions, computed in a first sample (training sample), must be tested in a new independent sample (test sample) [69,70].

### 3.2. Overall Strategy for Testing the Triadic Neural Systems Model

**Figure 2:**
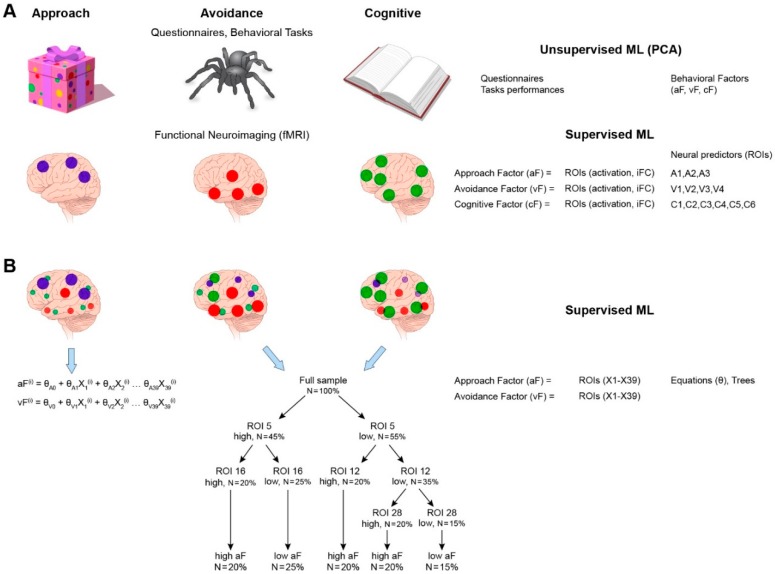
Strategy for Questions 1 and 2 to test the triadic neural systems model. All the data presented in this figure are totally fictitious. They only serve as concrete illustrations. The upper-panel addresses Question 1. Two sequential analyses are presented. (**A**) The first analysis is a principal component analysis (PCA is considered an unsupervised algorithm), which reduces and groups all behavioral data (from questionnaires, physiological, task performance) into latent factors. These factors map to the approach (aF), avoidance (vF), and cognitive (cF) domains. The second analysis applies a supervised ML algorithm. This algorithm uses the latent behavioral factors (aF, vF, cF) provided by the PCA as the predicted (output) data. The input data are all the significant regions of interest identified in the neuroimaging scans (task-related activation: regions of interest (ROIs), and resting state connectivity: intrinsic functional connectivity (iFCs)). In the present example, the input variables are scalar variables. The solution of this algorithm reveals the brain regions (ROIs, iFCs) that best predict the behavioral factors (aF, vF, cF). We consider these brain regions to represent the networks underlying the coding for aF, vF, and cF. These networks consist of three regions for aF, A1–A3, four regions for vF, V1–V4 for vF, and six regions for cF. These networks are drawn on the brain illustrations for concrete illustration. However, their location is not to be interpreted, since they are arbitrary. (**B**) The lower-panel delineates the analytic path to identify the relative contributions of the three neural networks of approach, avoidance, and cognitive (provided by the solution of Question 1) to individual behavioral propensity. This path rests on a supervised ML algorithm. The input variables consist of all neural predictors isolated in Question 1, but extracted from the three task-related fMRI scans, i.e., in the three behavioral contexts of interest. Therefore, 13 brain regions are extracted from each of the reward, aversive, cognitive task-related scans, making up 39 input variables. X1 is the extracted value of ROI1. There are 39 ROIs, and thus 39 X’s. The superscript ^i^ corresponds to the subject i. The predicted variables (output variables) are the latent behavioral factors (aF, vF) calculated in Question 1. The cognitive factor is not examined because, presently, the triadic model is specifically focused on modeling approach and avoidance behaviors, and their relative dominance in the behavioral patterns of individuals. The solution of the ML algorithm provides equations and trees. The equations assign weight to each predictor (parameters that are unique to the equation predicting aF and those predicting vF). The trees permit to assess how the different variables interact to predict the behavioral factors (aF, vF).

The triadic neural–systems model can be tested in three sequential stages to address three main aims (questions) that seek to delineate: (1) the functional architecture of each system: describe and validate the brain mapping of each system (the approach, avoidance, and control system); (2) the dynamic interaction among the three systems: describe and validate how these systems work together to generate adaptive motivated actions; (3) the maturation of the triadic model: model how these three systems and their interactions develop with age. The strategy to test the first two questions is illustrated in Figure 2.

To accomplish these three aims, three types of data need to be collected in healthy individuals. These types of data include measures of (1) functional neural architecture, (2) behavioral characteristics, and (3) changes of neural and behavioral measures with maturation. Furthermore, each of these sets of data should be acquired in three different contexts, an appetitive context for approach responses, an aversive context for avoidance responses, and a cognitive context for control responses. 

The first two questions do not require pediatric samples. Although it would be optimal for the samples of Question 1 and Question 2 to be independent, it is not a requirement. The samples should be large enough to apply machine learning analysis, allowing for a larger subsample of >150 subjects to be used as a training sample to define the model, and a smaller subsample of >50 subjects to be used as the testing sample to validate the model. For Question 3, an optimal design would be that of a longitudinal study of a large community sample of children (e.g., *n* > 300). Research consortia such as ABCD, NCANDA, IMAGEN, and connectome [59,60,61,62] are currently collecting longitudinal behavioral and neuroimaging measures in large pediatric samples. Since these data are made available to the public, the present discussion is highly propitious. 

### 3.3. Question 1: Functional Architecture of Each System

Question 1 aims at defining the networks that are associated with the generation of three domains of behavior: approach, avoidance, and cognitive control. Two types of data were used for this goal: behavioral and neural measures. The strategy to test Question 1 progressed in two steps for data organization and for the computation of the predictive model (Figure 2). 

#### 3.3.1. Data Organization/Reduction

**Figure 3:**
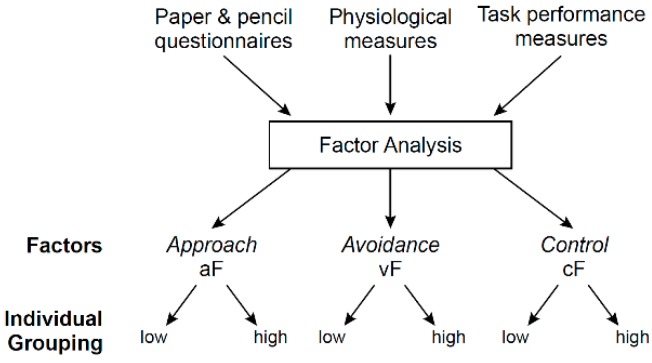
Principal component analysis (PCA) for behavioral data organization and reduction and for addressing Question 1, step 1. This step pools together all behavioral variables that are entered into a PCA. The PCA is aimed at grouping the behavioral variables into components (factors) based on their intrinsic properties. Based on the nature of the input variables, it is most likely that the variables will be groups into at least 3 factors, one capturing approach, another avoidance, and a third cognitive control. Other factors may also emerge. But, for simplicity, we only consider the three factors of interest, aF, vF, and cF for the approach, avoidant, and cognitive factors, respectively. Subjects can then be divided by their score level, e.g., high-score group versus the low-score group. Of note, the number of groups is arbitrary, since subjects could as well be separated into three groups, for example, based on low, moderate, and high factor scores.

The behavioral measures were expected to be numerous, including paper and pencil measures (self-report, interviews), task performance in both clinic and MRI (cognitive, motivation, emotion tasks), and physiological data (e.g., skin conductance, heart rate, EMG) (Figure 3). Therefore, a first step was to organize and reduce these measures to isolate latent factors most representative of the three domains of approach, avoidance, and control. In other words, rather than dictating which behaviors and questionnaires are associated with any one of the triadic systems, a data-driven approach can yield more objective and valid categorization of behavioral data. Principal component analysis (PCA) [71] is a reasonable approach to map existing behavioral data to distinct functional domains. This way, many metrics (e.g., questionnaire ratings, task performance, stress physiological responses) can be employed to reveal clusters (factors) of behavioral items that best describe the three behavioral domains of approach, avoidance, and control. Principal component analysis identifies the factors (eigen vectors) that best capture the variance in the existing dataset. These factors can be arranged in the order of their importance as defined by their corresponding eigen values. Ideally, a PCA approach would identify three principal factors that account for almost all of the variance of the behavior-based dataset, and which would map to the approach (aF), avoidance (vF), and cognitive control (cF) behavioral domains. 

The results of the PCA might be more complex. This analysis could provide more than one factor score for each of the three behavioral domains. In addition, other factors, not related to the triadic domains, could emerge. Finally, the possibility for not being able to clearly identify an approach, avoidance or cognitive factor also exists, although the nature of the inputted items, all related to the behaviors of interest, make this possibility unlikely. For simplicity, only one behavioral factor for each domain will be used as illustration, and each factor score can be used to define two groups, one with individuals scoring low on the given behavioral factor, and the other group scoring high. Finally, additional unrelated factors, which can also emerge from the PCA results, will not be discussed here. The next step was to identify which and how neural measures predict each behavioral domain, respectively. 

#### 3.3.2. The Predictive Model

**Figure 4:**
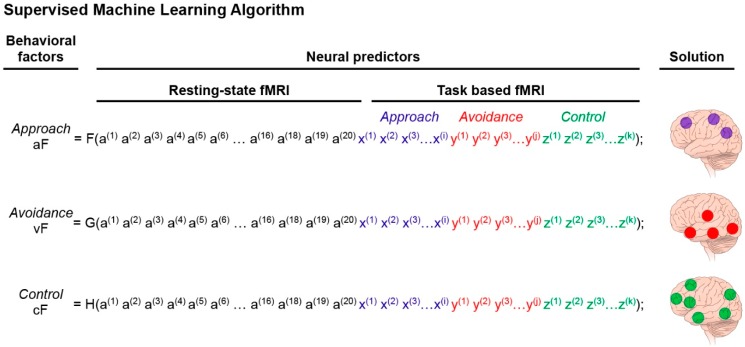
Supervised machine-learning step. Supervised ML analysis to identify the networks predicting the three PCA factors of interest (aF, vF, cF). This algorithm computes the predictive value of the extracted neural measures. As such, it provides the components (nodes) of the neural networks that predict the behavioral factors previously identified by PCA (Figure 3). The predictive model can take the form of equations, F for aF, G for vF, and H for cF. The predictors are neuroimaging data. More specifically, a ^(1)^ is the first extracted value of the resting state functional magnetic resonance imaging (fMRI), up to a^(20)^ which is the value of the last extracted measure that passes the significant threshold set for the resting state study (this number is arbitrary). The variable x^(1)^ is the extracted value of the most significant regional activation of the approach-task-based fMRI, and (i) is the number of regions extracted from the reward-task fMRI scans; y^(1)^ corresponds to the avoidance domain with (j) being the number of regional activations extracted from the avoidance-task based fMRI scans; and z^(1)^ corresponds to the control domain with (k) being the number of regional activations extracted from the control-task fMRI scans. The brain illustrations on the right of the equations represent the solution of the ML analysis, which identifies the nodes (neural predictors) that best predict aF, vF, and cF, respectively (i.e., have the highest weight). The number of these regional activations (or nodes) is arbitrarily set to three for the approach factor, four for the avoidance factor, and six for the control factor. These nodes define the neural networks that best predict reactivity to the behavioral domains of the triadic model.

This step identifies the activity patterns of the neural networks predicting each of the three behavioral domains, quantified by the data reduction PCA step as factors: approach factor (aF), avoidance factor (vF), and control factor (cF). These behavioral domains are examined separately, but concurrently, using the same procedure. This step uses supervised ML tools. The ML algorithms (such as support vector machine model or random forest model) implement iterative computations to train the computer to recognize the neural patterns that optimally predict the behavioral factor scores (aF, vF, and cF). In other words, the goal of these analyses is to produce the most efficient (low-cost, low-noise) predictive model of a given behavior using neural activity patterns as predictors. The neural inputs (i.e., predictors) to the model consist of resting state connectivity (iFC) measures, in the form of weights between hubs (e.g., ventral striatum to ventromedial prefrontal cortex), and regional blood-oxygen-level dependent (BOLD) activations during task-related fMRI (Figure 4). These input data are scalar, since a single variable (beta for activation, or Fisher-transformed Pearson correlation for connectivity) is extracted, and not the timeseries of BOLD activity/connectivity data. One advantage of ML methods is that multiple neural variables can be examined as predictors of a given behavior in a single model, without violating the assumptions of the statistical model (e.g., *t*-tests are conducted with the assumption that the data is normally distributed). In Figure 4, we present three equations for each behavioral domain (F, G, H). Each equation is unique to its behavioral domain in terms of the parameters (computed by the ML algorithms) that weigh the neural predictors. A higher weight signifies a more determinant role in predicting the behavior. The dominant predictors are those that also appear most determinant in decision trees (see comment below describing decision trees in more details), which maximize the output separation (subjects scoring high on the behavioral domain versus those scoring low). Furthermore, many ML models, by default, test interactions. For example, classification trees are a type of machine-learning model where the sample is partitioned in a sequence of steps (e.g., two steps: tree sequentially split by gender and then by family history of substance use). 

We provide for the reader a comment, which describes decision trees in more details. “*A decision tree is s supervised learning algorithm, which attempts to classify or regress a dataset in a hierarchical fashion. As the name suggests, a decision tree has a tree-like architecture, with points (nodes) at which the tree splits into branches (edges). The split in the tree depends on the dimension that best separates the data. The criterion governing this split is defined by a cost function. The cost function computes the difference between the predefined label/score assigned to the datapoint and the prediction based on the split. The dimension, along which the cost function is minimized, becomes the node criterion. This process is then repeated in a recursive fashion, with the ends of the new branches becoming new nodes. A popular algorithm used in decision trees is the Recursive Binary splitting algorithm, where each node splits into 2 branches in a recursive fashion. An important consideration while using decision trees is to know when to stop the tree from growing any further and which splits to remove. A number of thumb rules can be used For example, one can predefine the maximum possible depth (length of the longest path) of the tree; or pre-set the minimum number of datapoints required in a branch before a split can occur; or get rid of the branches that do not impact the cost function too much.*”

**Figure 5:**
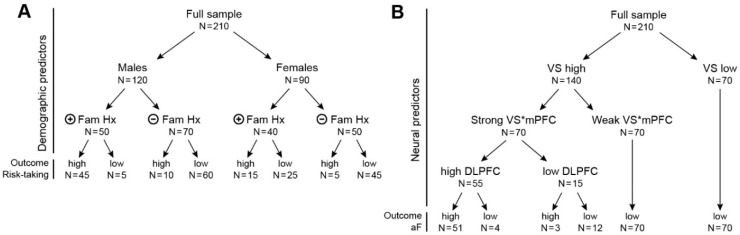
Two fictitious examples of solutions using classification tree algorithms. (**A**) Example 1 illustrates a ML classification of subjects into groups of high or low risk-takers (high versus low value of the outcome measure), in function of sex and family history of substance use (fmhx-su). The sample is composed of 120 males and 90 females. The modulation of risk-taking by fmhx-su is different in males and females. Males with a positive fmhx-su are more prone to be high risk-takers (45 of 50, 90%) compared to males without fmhx-su (10 of 70, 14%). However, this factor does not seem to be as determinant in females, 15 of 40 females (38%) compared to 5 of 45 (11%) without fmhx-su. (**B**) Example 2 illustrates a ML classification of individuals as a function of high versus low propensity for approach behavior (high aF versus low aF) using neural predictors. This tree shows that the ventral striatum (VS) is the strongest predictor of high aF. All the individuals with low VS have a low aF scores. In contrast, the association of high VS sensitivity with high aF is modulated by the connectivity of VS* the medial prefrontal cortex (mPFC), and by the activity of the dorsolateral prefrontal cortex (DLPFC). This tree clearly illustrates how such analysis can help clarify interactions among neural predictors of specific behaviors. VS = ventral striatum activation, VS*mPFC = intrinsic connectivity between VS and medial prefrontal cortex, DLPFC = dorsolateral prefrontal cortex.

We present two simple fictitious examples of classification tree results in Figure 5. The first example (Figure 5A) uses demographic and clinical data to classify participants as high or low risk-takers. The model places gender at the top of the tree, i.e., first key variable that splits the sample in function of the propensity for risk-taking. It shows that males are more frequently high risk-takers than females. The second variable emerging as the next strongest predictor is family history of substance use. A positive family history of substance use strongly increases propensity for risk-taking in males, but less so in females. In other words, a positive family history confers risk that differs between males and females. 

The second example (Figure 5B), also totally fictitious, is closer to the thematic of this paper, i.e., identify neural predictors of behavior. In this made-up example, the classification tree analysis reveals that approach behavior (e.g., aF) is most frequently (at the top of the tree) predicted by high ventral striatal (VS) response to reward, but only if the circuit’s strength between VS and ventral medial PFC (vmPFC) is low, and the dorsolateral PFC (DLPFC) response is high. Machine learning would be able to identify this model, but linear models would not, unless the interaction term was specified (known a priori). With many potential interactions, it would be challenging for human researchers to consider all the possible interaction terms, but it is easy for a machine. Ultimately, a classification tree analysis would delineate the brain regions that matter in the modulation of approach behavior. In the theoretical example of Figure 5B, the most powerful predictors are the VS, the vmPFC, and the dorsolateral PFC (DLPFC) (all fictitious examples).

#### 3.3.3. Output of the Predictive Model of the Characterization of the Three Neural Systems

Taken together, the expected output of Question 1 is the delineation of the main nodes engaged (via activation or connectivity) in each of the three functional domains. In addition to providing the identity of these nodes, Question 1 also reveals patterns of interactions that predict the degree of behavioral propensity towards either approach, avoidance or control. Here, these patterns of interactions are not used in the subsequent analyses, although they are important to fully characterize the neural substrates of each behavioral domain. Analyses for Question 2 will focus on the main nodes identified in Question 1. For illustration, we will arbitrary assign nodes to each neural system: approach system, A1, A2, A3, avoidance system, V1, V2, V3, V4, and control system, C1, C2, C3, C4, C5, C6. In actuality, some nodes are likely to be common to two or all three systems, but, for simplicity, this situation is not considered. 

### 3.4. Question 2: Dynamic Interactions among the Three Systems

Question 2 concerns how the neural predictors of all three behavioral domains (i.e., approach A1–A3, avoidance V1–V4, and control C1–C6) work together in each of the appetitive and aversive contexts. Indeed, the triadic model predicts that, in an appetitive context, the approach system will be active and more tightly intra-connected, whereas the avoidance system will tend to be silenced [7,72]. The opposite pattern would characterize the neural pattern in an aversive context (Figure 1). The control network would manifest different couplings with the approach versus avoidance system as a function of the appetitive versus aversive context. Clearly, the reality is more complex, particularly with expected interactions among nodes of the different neural systems, which we anticipate being able to characterize using ML strategies. 

#### 3.4.1. Question 2, Step 1

**Figure 6:**
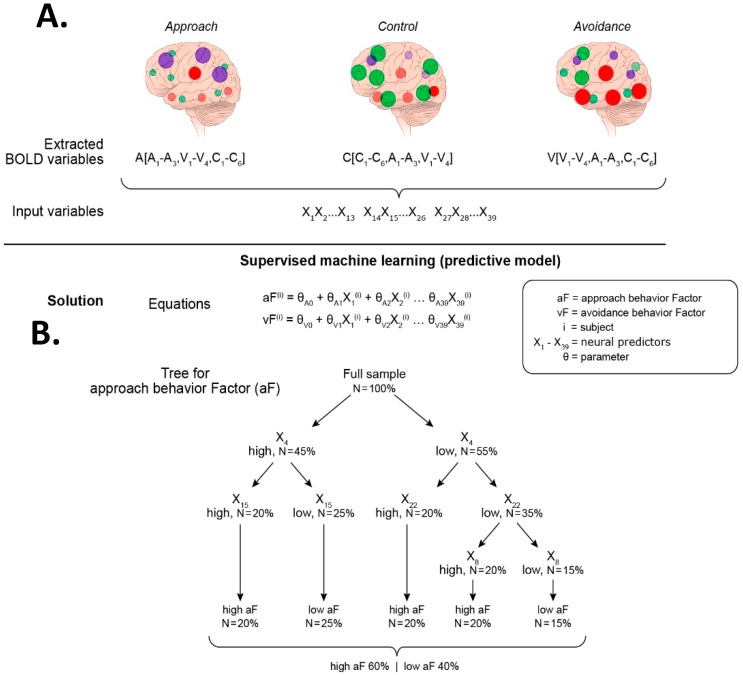
Strategy for addressing Question 2, Steps 1–2. Extraction of neural predictors and supervised ML to predict behavioral factors aF and vF. (**A**) Step 1: Neural data extraction. This step consists of gathering all potential neural predictors of the behavioral factors identified in Question 1 (Figure 4). These neural predictors (nodes) are now all extracted from every task-based fMRI scan (cognitive-task fMRI, avoidance-task fMRI, and approach-task fMRI). The brain illustrations show the activation maps of all the nodes of interest, the approach nodes (purple), the avoidance nodes (red), and the control nodes (green), in each task-based fMRI scan. The extraction of the blood-oxygen-level dependent (BOLD) signals for all regions of interest (ROIs) (13 per scan) are labelled X_1_ through X_39_. (**B**) Step 2: Supervised ML analysis. The extracted 39 ROIs variables (X_1_–X_39_) are the predictors used in two analyses, one for the approach domain and the other for the avoidance domain. Each analysis can be performed using two supervised algorithms. The first algorithm uses a linear model to estimate the strength of each neural predictor (theta weights). The second algorithm uses a decision tree to provide a hierarchical structure that informs more directly the interactions among neural predictors. The example shows the sample divided in two groups, one group with low aF scores, and the other group with high vF scores. The tree depicts four ROI with significant weights in predicting the groups. These ROI are ROI_4_, ROI_15_, ROI_22_, and ROI_8_, and their respective BOLD values are X_4_, X_15_, X_22_, and X_8_.

The neural predictors identified in Question 1 provide the basis for the input variables of this analysis (Figure 6). As a reminder, the fictitious results of Question 1 reveal three brain regions (A1–A3) that predict approach, four brain regions (V1–V4) for avoidance, and six brain regions (C1–C6) for control. The strategy to query Question 2 takes two steps: (1) the collection of all neural data from all three contexts (approach, avoidance, control), and (2) a supervised ML “categorization” analysis (Figure 6).

The first data collection step (Figure 6A) is a re-examination of the task-based fMRI scans, in order to extract the values of the neural predictors of all three contexts pooled together. In other words, the “approach” nodes (or regions of interest, ROI) A1–A4 are also extracted from the control- and avoidance-related scans, the “avoidance” ROIs V1–V3 from the control- and approach-related scans, and the “control” ROIs C1–C5 from the avoidance- and approach-related scans. Therefore, the number of potential neural predictors for Question 2 is tripled (3 approach + 4 avoidance + 6 control ROIs = 13 ROIs, whose activity is extracted from the reward-task fMRI + the negative-task fMRI + the cognitive-task fMRI, i.e., 13 × 3 = 39). Similar to Question 1, for simplicity, only scalar variables are used as input data, not timeseries. 

#### 3.4.2. Question 2, Step 2

In the second step (Figure 6B), the input data (*n* = 39) are categorized based on their predictive value of each behavioral domain. This analysis falls under the supervised ML approach, using learning algorithms of support vector machines or decision trees, as described in Question 1. To avoid confusion, the supervised ML algorithm, used in Question 1, identified the neural predictors from task-based and resting-state scans of approach, avoidance or control propensity. In Question 2, this ML algorithm is used to identify the neural predictors from the pooled neural predictors (*n* = 13 extracted from all three contexts, *n* = 13 × 3) of the propensity for approach, avoidance, and control separately. The result of this last analysis informs the patterns of interactions across the three neural systems that support the behavioral domains of the triadic model. These results would represent a major advance in knowledge of how brain systems work together to organize behavior. 

Figure 6 illustrates two types of representations of the results. The equations provide measures of the strength of each neural predictor (theta weights). The trees provide a representation of the hierarchical power of the neural predictors to influence behavioral outcomes, informing more directly interactions among neural predictors. 

### 3.5. Question 3: Maturation of the Triadic Neural System Dynamics 

**Figure 7:**
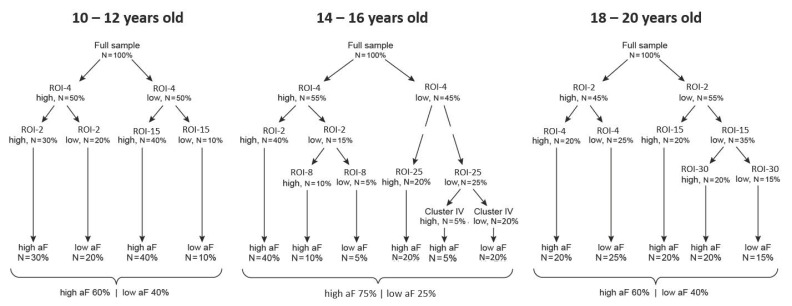
Decision trees across development. Fictitious examples of developmental analysis of neural predictors of behavior: examples of decision-trees at each follow-up. Changes in classification trees at different age groups inform the evolution of mechanisms underlying motivated behaviors of approach and avoidance.

The strategy for Question 3 is a simple replication of the steps described above, but at each follow-up (e.g., 12 years old, 16 years old, and 20 years old). An example of a possible outcome is presented in Figure 7, which clearly shows how this process could inform the developmental trajectories not only of the triadic neural systems, but also of their interactions. We summarize the steps below:

(a) Step 1: Behavioral characterization. The predicted behavior outcome measures are computed as in Question 1 by conducting a factor analysis of all available behavioral data, at each follow-up.
(b) Step 2: Brain-behavior classification: This step gives rise to two types of results: trees that depict the hierarchical organization of predictors at each follow-up point and equations that quantify the contribution of each cluster to a given behavioral domain, respectively.

## 4. Conclusions

In this paper we offer a preview of the potential contribution of the latest, so far most powerful, tools to help delineate how brain networks code motivated behaviors. This window into brain-behavior causal mechanisms can have invaluable implications for understanding developmental changes in health and diseases, and can provide critical guides for refining and extending research in this field. Specifically, the present work provides a guide that can serve to query the mechanisms underlying the contributions of prefrontal circuits to emotion regulation. Finally, findings that emerge from such an analytical approach can raise key questions that, in turn, can be examined in focused studies, or warrant a re-examination of previous data.
